# Sophomore nursing students’ perception of their Professional Behavior toward Rehabilitation patients: a cross-sectional study

**DOI:** 10.1186/s12912-023-01616-y

**Published:** 2023-11-23

**Authors:** Anat Amit-Aharon, Sigalit Warshawski

**Affiliations:** Department of Nursing, Steyer School of Health Professions, Faculty of Medicine, Tel Aviv University, Tel Aviv, Israel

**Keywords:** Nursing students, Rehabilitation nursing, Professional behavior

## Abstract

**Background:**

Rehabilitation is considered one of the elements of universal health coverage, emphasizing its importance for every person in need throughout the life course. Nurses play a pivotal role in the rehabilitation team as they possess the competencies to help individuals manage health problems and maximize potential well-being. Yet, little is known regarding knowledge of this subject among nursing students, as well as regarding their attitudes, thoughts, and professional behavior. This study aimed to explore: (a) Sophomore students’ attitudes, feelings, thoughts, and professional behavior toward rehabilitation patients; and (b) Differences in the research variables as affected by students’ previous exposure to rehabilitation patients.

**Methods:**

A cross-sectional survey design among sophomore nursing students. A questionnaire was distributed through a commercial internet survey provider (Qualtrics.com) before the beginning of a mandatory course on “nursing rehabilitation”, introduced for the first time in 2022 in all Israeli universities. Students were divided into three groups according to their previous exposure to rehabilitation patients: no professional or personal previous exposure to rehabilitation patients; previous professional or personal exposure to rehabilitation patients; and previous exposure to rehabilitation patients both in one’s professional and personal life. The study adhered to the STROBE guidelines.

**Results:**

The sample consisted of 122 participants. Significant differences were found between the groups by their previous exposure to rehabilitation patients. Participants with no previous exposure to rehabilitation patients were found to have lower self-perceived capability to care for rehabilitation patients and more negative professional behavior toward rehabilitation patients and their families compared to the other two groups (*H =* 22.06, *p* = 0.006, *H* = 23.86, *p* = 0.03, respectively). No other statistical differences were observed between the groups.

**Conclusions:**

The findings emphasize the importance of exposing students to the field of nursing rehabilitation and to the care of rehabilitation patients. Exposure to nursing rehabilitation specifically during one’s studies, through theoretical learning and clinical experience, can promote positive attitudes, professional values, and positive professional behavior toward these patients and their families.

**Supplementary Information:**

The online version contains supplementary material available at 10.1186/s12912-023-01616-y.

## Background

Rehabilitation is considered one of the elements of universal health coverage (UHC), meaning that rehabilitation services must be available worldwide for every person in need [1]. The term ‘rehabilitation’ refers to a targeted and time-delimited process that involves collaboration between multidisciplinary professionals, the patient, and the patient’s family or caregivers. Rehabilitation aims at improving and maintaining the functioning of persons with health conditions defined as acute, chronic, impairment, or injury that limit functioning, as well as persons with disabilities [2, 3]. Rehabilitation must be available in almost all healthcare settings and all phases of care [4]. To achieve this, rehabilitation provides a set of interventions addressing individual needs and requires a multi-professional rehabilitation team approach [5]. Nurses play a pivotal role in rehabilitation teams. According to the definition by the International Council of Nurses (ICN, 2020), “Nursing encompasses autonomous and collaborative care of individuals of all ages, families, groups, and communities, sick or well, and in all settings. Nursing includes promoting health, preventing illness, and caring for ill, disabled, and dying people” [6]. Accordingly, nurses possess a holistic approach to caring and are natural members of the multidisciplinary rehabilitation team. They are and can be the main actors in fulfilling patients’ primary and constantly more complex and ever-changing needs.

The Association of Rehabilitation Nurses (ARN 2014) states that “rehabilitation nursing is precisely the response to functional impairments and deterioration of individuals” [7]. Since the 1970s, rehabilitation nursing has become a particular sector of the nursing spectrum. The central role of nurses can also be highlighted through the different stages of nursing rehabilitation that take place in various healthcare environments: acute rehabilitation, post-acute rehabilitation, nursing homes and geriatric care, long-term rehabilitation with a particular focus on persons with disabilities that is often performed in the person’s home, and so-called community-based rehabilitation services (CBR) [4].

In September 2020, for the first time, the Israeli Ministry of Health recognized four nurses as practitioner nurses in rehabilitation. In March 2021, the Director of Nursing at the Ministry of Health published guidelines for the authorization of nurse specialists in rehabilitation. The guidelines stated: “The nurse specialists in rehabilitation is a key professional figure who helps people deal with long-term health problems, reduces dependency, and maximizes potential” [8]. This constituted formal recognition of the significant role of the nursing profession in the rehabilitation treatment process. Subsequently, the Director of Nursing added, a course in “Nursing Rehabilitation” consisting of 14 semester hours to all core undergraduate nursing programs, for the first time. The course is given in the second year of the four-year program and focused primarily on providing nursing students with up-to-date and basic knowledge of the principles of rehabilitation treatment. These changes in the nursing curriculum and the recognition of rehabilitation as a clinical specialty raise a question regarding students’ attitudes toward rehabilitation care and their feelings while caring for rehabilitation patients.

Studies conducted in the UK, Denmark, Finland, and Norway among nurses employed in rehabilitation facilities revealed that nurses thought that their pre-licensing education had not provided them with adequate knowledge and skills to prepare them for their role in rehabilitation. Pre-registration education provided only a basic introduction to rehabilitation. This contrasted with acute nursing care, for which pre-registration studies provide a good background and preparation [9, 10]. A study in Taiwan emphasizes the knowledge and skill gaps between nurses working in general internal medicine wards and in chest medicine wards regarding the promotion of pulmonary rehabilitation on the wards. Differences in pulmonary rehabilitation knowledge, attitudes, and skills were noted between the two groups, in favor of the nurses working in chest medicine wards [11].

Regarding nursing students, the literature review revealed a paucity of studies on the subject worldwide, where the existing research focuses mainly on students’ attitudes toward people with disabilities rather than specifically on rehabilitation. It is important to note that most of the studies were conducted about two or three decades ago. For example, Thompson et al. (2003) explored whether a change in curriculum and clinical experience affected nursing student attitudes toward disabled persons. Students’ attitudes were significantly more positive after completing their senior year. The findings suggest that education and exposure to individuals with disabilities positively affected nursing students’ attitudes toward individuals with disabilities [12].

It may be concluded that nursing plays a vital role in the therapeutic process of rehabilitation patients. However, nurses lack formal academic knowledge of rehabilitation. Considering the current gap in the literature regarding the treatment of rehabilitation patients, the present study examined attitudes, feelings, thoughts, and professional behavior toward rehabilitation patients among nursing students, in preparation for an academic course on the role of nursing as part of a multi-professional team caring for rehabilitation patients.

## Methods

### Aims

The aim of the study was to explore: (a) Sophomore students’ attitudes, feelings, thoughts, and professional behavior toward rehabilitation patients; and (b) The differences in the research variables as affected by students’ previous exposure to rehabilitation patients.

### Research design and setting

The current study utilized a descriptive, cross-sectional design. The study adhered to the STROBE guidelines.

### Sample

All second-year undergraduate nursing students in the Baccalaureate and Accelerated programs at a major Israeli university (245 students) were invited to participate in the study. Second-year students in the two programs were selected for participation in this study since they had already completed at least two clinical placements in acute care settings in hospitals and experienced basic nursing care interventions.

The sample size was calculated using G*Power software [13]. To conduct an ANOVA, the following parameters were inserted: medium-large effect size of 0.30, α = 0.05, power = 0.80, and number of groups = 3. The minimum sample size calculated was 111 participants. Hence, the 122 participants in the current study should be satisfactory. Nevertheless, after the data was collected, the statistical tests for approving the normal distribution of the data were not confirmed. Therefore, it was decided to perform non-parametric tests. According to the literature [14], non-parametric tests are the most valuable and effective for small samples; it was assumed that the determined sample size (n = 122) is sufficient for the Kruskal-Wallis test.

### Instrument

The research instrument was a structured questionnaire including three sections:

*A) Participants’ sociodemographic data and previous exposure to rehabilitation patients.* Sociodemographic data included age, gender, country of birth, ethnicity, and marital status. Two additional items assessed the participants’ prior exposure to rehabilitation patients: (1) Have you previously cared for a rehabilitation patient? (yes/no); (2) Do you have a relative undergoing a rehabilitation process? (yes/no)

*B) Attitudes toward rehabilitation patients and rehabilitation nursing.* The research team developed this section, inspired by the capability, opportunity, and motivation behavior (COM-B) framework [15]. The COM-B framework refers to self-assessment of behavior regarding capability, opportunity, and motivation to engage with complex issues [15], such as rehabilitation patients and nursing. Loft et al. (2018) used the COM-B framework among nurses to assess nurses’ self-perceived capability, opportunity, and motivation to care for stroke rehabilitation patients before and after training in an acute stroke unit. The current research questionnaire was developed to assess students’ attitudes toward rehabilitation patients and rehabilitation nursing. The questionnaire included 21 items in three sub-scales:

1. Perceived physical and psychological capability to care for rehabilitation patients included the knowledge and skills required to work with these patients, for example: “I know how to work according to the principles of rehabilitation nursing”. Participants were asked to rank their agreement regarding each item on a 5-point scale from 1 (don’t agree at all) to 5 (strongly agree). A higher score means higher perceived capability to care for rehabilitation patients. The internal consistency based on Cronbach’s alpha was 0.86.
2. Perceived professional opportunity in rehabilitation nursing, including the cognitive and emotional process needed for analytical decision making. For example, “Treating a patient with a rehabilitation potential is a professional challenge for me.” Responses were given on a 5-point scale from 1 (don’t agree at all) to 5 (strongly agree). A higher score means a higher perception of the professional opportunity in rehabilitation nursing. The internal consistency based on Cronbach’s alpha was 0.73.
3. Students’ motivation to care for rehabilitation patients. This means an external factor that motivates the students and makes rehabilitation care a possible behavior. A sample item is, “I am proud of the contribution of nursing in the field of rehabilitation.” Responses were given on a 5-point scale from 1 (don’t agree at all) to 5 (strongly agree). A higher score means higher motivation to care for rehabilitation patients. The internal consistency based on Cronbach’s alpha was 0.72.

*C) Students’ feelings, thoughts, and professional behavior toward rehabilitation patients*: This section of the questionnaire was adopted from the validated Multidimensional Attitudes Scale (MAS) for people with disabilities [16]. The section began with a short vignette followed by three sub-sections. The first deals with the feelings the vignette raised (the original Cronbach’s alpha was 0.90), the second with cognitive thoughts (the original Cronbach’s alpha was 0.88), and the third with behavior (the original Cronbach’s alpha was 0.83). This method used an indirect approach to measure feelings, thoughts, and behavior referents [16].

The original questionnaire was adapted to the present study, and the vignette dealt with a nurse caring for rehabilitation patients in a hospital. The vignette presented the participants with a short case description of a rehabilitation patient hospitalized in a rehabilitation unit after experiencing a stroke. The patient was described as having a low rehabilitation potential. His family visits him frequently and demands that the attending nurse and physician not stop the rehabilitation process. Immediately following, participants are asked to reply to three sections based on the earlier description: (a) possible feelings of the nurse in the vignette toward the patient, (b) possible thoughts of the nurse in the vignette toward the patient, and (c) possible professional behaviors performed by the nurse in the vignette concerning the patient (Supplementary material 1).

1. Feelings - This section contains a list of 13 feelings (for example rejection, fear, empathy for the patient, empathy for the family). Participants were asked to rank the likelihood that the nurse would feel each of the feelings toward the patient, on a 5-point Likert scale from 1 (not likely at all) to 5 (very likely). Factor analysis of this part indicates that it is divided into two sub-scales: negative feelings (Cronbach’s alpha = 0.87, where a higher score means more negative feelings toward the patient) and positive feelings (Cronbach’s alpha = 0.84, where a higher score indicates more positive feelings toward the patient and family).
2. Thoughts - This section contains a list of five positive thoughts the nurse in the vignette may think regarding the patient (for example: “I have to take good care of that patient,” or “I must talk to the family and explain to them the patient’s condition”). Participants are asked to rank the likelihood that the nurse would think each of the thoughts toward the patient, on a 5-point Likert scale from 1 (not likely at all) to 5 (very likely). A higher score means more positive thoughts regarding the rehabilitation patient. Cronbach’s alpha = 0.70.
3. Professional behavior – This section contains a list of five negative professional behaviors the nurse in the description might perform (for example: “I will ignore the family” or, “I will find an excuse to transfer the patient’s care to another nurse”). Participants are asked to rank the likelihood that the nurse would perform each of the professional behaviors toward the patient, on a 5-point Likert scale from 1 (not likely at all) to 5 (very likely). A higher score means more negative professional behavior toward the patient. Cronbach’s alpha = 0.78.

The whole questionnaire underwent content validation by two senior rehabilitation practitioner nurses. Each one of them examined the questionnaire alone and later a joint meeting was held with the research team. A discussion was conducted, and the vignette and the items were corrected with complete agreement.

### Procedure

All second-year undergraduate nursing students in the university nursing program were approached two weeks before the “Nursing rehabilitation” course commenced, during March-June 2022. The study was conducted using the format of a commercial internet survey provider (Qualtrics.com). A link to the online questionnaire appeared on a short explanatory page that presented the research aims. The page was posted in a social media group for second-year students, by a research assistant who was not an instructor of these students. The study received the approval of the university’s ethics committee (#0004421-1). A link to the Qualtrics code was sent to all sophomore students via their closed media groups. The link to the online questionnaire appeared on a short explanatory page that clarified the research purposes. Participants were assured that responses to the questionnaires would be anonymous and that their confidentiality would be maintained. Consent was assumed by submission of the questionnaire. Only students who agreed to participate and gave their informed consent could access the questionnaire.

### Data analysis

Based on the two questions regarding previous exposure to rehabilitation patients, the participants were divided into three groups as follows: no previous exposure recoded = 0, defined as group A; previously treated rehabilitation patients or having known relatives in the rehabilitation process recoded = 1, defined as group B; both previously treated rehabilitation patients and having known relatives in the rehabilitation process recoded = 2, defined as group C. Descriptive statistics were used to compare the sample characteristics of the three groups.

The Kruskal-Wallis *H* test was conducted to explore the differences in the variables among the three groups. Pairwise comparisons were performed using Dunn’s procedure (for the post hoc test) with Bonferroni correction for multiple comparisons, and adjustment *p*-value was calculated. The data were analyzed using the SPSS-27 statistical package (SPSS Inc., Chicago, Ill., USA). Statistical significance was considered at *p* < 0.05.

## Results

Altogether, 161 questionnaires were returned, but 39 were excluded as they had not been fully completed (response rate 49.8%). Most of the participants were female (83.1%), with a mean age of 26.5 (SD = 6.06), Jewish (80.0%), and secular (63.8%) (Table 1). The participants’ characteristics according to the three research groups describes in Table 1: Group A, whose participants had no previous exposure to rehabilitation patients (56.3%); Group B, whose participants had previously treated rehabilitation patients or had relatives in the rehabilitation process (30.3%); and Group C, whose participants had both treated rehabilitation patients and had relatives in the rehabilitation process (13.4%). There were no statistically significant differences between the three groups, with the exception that participants in Group C were more inclined to have been born outside Israel and were older than those in Groups B and A.

**Table 1:** Participants’ characteristics

Variable	Entire sample	Group A No previous exposure to rehabilitation patients	Group B Previously treated rehabilitation patients or have known relatives in the rehabilitation process	Group C Both previously treated rehabilitation patients and have known relatives in the rehabilitation process		
	n (%)	n (%)	n (%)	n (%)	χ^2^	*p=*
Sample size*	122 (100)	67 (56.3)	36 (30.3)	16 (13.4)		
Gender					0.81	0.66
Male Female	19 (15.9) 99 (83.1)	12 (17.9) 55 (82.1)	4 (11.4) 31 (88.6)	3 (18.8) 13 (81.3)		
Place born					10.67	0.005
Israel Outside Israel	101 (84.9) 12 (10.1)	60 (92.3) 5 (7.7)	32 (94.1) 2 (5.9)	9 (64.3) 5 (35.7)		
Ethnicity					0.18	0.91
Jewish Arab	94 (80.0) 25 (20.0)	52 (77.6) 15 (22.4)	29 (80.6) 7 (19.4)	13 (81.3) 3 (18.8)		
Religiosity					0.25	0.88
Secular Traditional-ultra orthodox	76 (63.8) 41 (34.4)	41 (63.1) 24 (36.9)	24 (66.7) 12 (33.3)	11 (68.8) 5 (31.2)		
Marital status					4.17	0.12
Yes No	58 (48.7) 23 (19.3)	32 (47.8) 35 (52.2)	14 (47.2) 19 (52.8)	12 (75.0) 4 (25.0)		
Study program					4.79	0.09
Generic + premilitary Acceleration	73 (61.3) 46 (38.7)	45 (67.2) 22 (32.8)	22 (61.1) 14 (38.9)	6 (37.5) 10 (62.5)		
	**M (SD)**	**M (SD)**	**M (SD)**	**M (SD)**	**F**	***p=***
Age	26.5 (6.06)	25.6 (5.36)	26.8 (6.38)	29.8 (7.17)	3.20	0.04

*Totals may not add up to 100% because of missing data

A Spearman correlation test was conducted to explore the association between the research variables (Table 2). The main finding is a medium positive correlation between negative feelings and negative professional behavior (r = 0.34, *p* < 0.001). This means that the greater the negative feelings rehabilitation patients evoke, the greater the negative professional behavior toward rehabilitation patients.

**Table 2:** Spearman correlation between the research variables

Variable	1	2	3	4	5	6	7
Self-competence attitude	1	-0.24**	0.14	-0.05	0.16	0.21*	-0.14
Knowledge gap perception		1	0.27**	0.17	0.16	0.08	0.07
Essential role of nursing attitude			1	0.11	0.33***	0.27**	-0.12
Negative feelings				1	0.11	0.04	0.34***
Positive feelings					1	0.34***	-0.14
Positive thoughts						1	-0.17
Negative professional behavior							1

*p < 0.05 **p < 0.01 ***p < 0.001

Table 3 presents the results of the Kruskal-Wallis test analysis. The Kruskal-Wallis test revealed that the groups’ median scores were statistically significantly different regarding their attitude to their capability to care for rehabilitation patients, H = 11.85, *p* = 0.003. Subsequently, post hoc pairwise comparisons using Dunn’s procedure with Bonferroni correction for multiple comparisons revealed statistically significant differences in the scores for participants’ attitude to their capability between Groups B and A. Group B participants had a statistically significantly higher capability to care for rehabilitation patients compared to participants in group A (Median. 3.16 vs. Median. 3.05, Adj. *p* = 0.006) but other group combination.

The Kruskal-Wallis test revealed that the median scores for negative professional behavior of participants in the different groups were statistically significantly different, H = 6.49, *p* = 0.03. Subsequently, post hoc pairwise comparisons using Dunn’s procedure with Bonferroni correction for multiple comparisons revealed statistically significant differences in the scores for negative professional behavior between Group A and Group C. Group A participants had statistically significantly higher scores for negative professional behavior compared to participants in Group C (Median 1.90 vs. Median 1.65, adj. *p* = 0.03), but no such difference was found for any other group combination.

No other significant differences were observed.

**Table 3:** Kruskal-Wallis test for group differences and Dunn’s procedure post hoc with Bonferroni adjustment

Variable	Group A No previous exposure to rehabilitation patients	Group B Previously treated rehabilitation patients or have known relatives in the rehabilitation process	Group C Both previously treated rehabilitation patients and have known relatives in the rehabilitation process			Dunn’s procedure post hoc with Bonferroni adjustment		
	N = 67	N = 36	N = 16	*H*	*p=*	*H*	Adj.*p=*	Post-hoc test
	Median	Median	Median					
Attitudes								
Capability Opportunity Motivation	3.05 4.00 4.48	3.16 3.77 4.36	3.22 3.80 4.66	11.85 3.18 2.25	0.003 0.20 0.32	22.06 - -	0.006 - -	B > A
Affect								
Negative feelings Positive feelings	2.81 4.60	2.79 4.60	2.81 4.80	5.14 1.78	0.07 0.41	- -	- -	
Positive thoughts	4.32	4.32	4.32	0.56	0.76	-	-	
Negative professional behavior	1.90	1.90	1.65	6.49	0.03	23.86	0.03	A > C

## Discussion

The current study aimed to explore sophomore nursing students’ attitudes, feelings, thoughts, and professional behavior regarding rehabilitation patients. The findings suggest that students with no previous exposure to rehabilitation patients have a lower self-assessment of their capacity to care for rehabilitation patients and higher negative professional behavior toward rehabilitation patients and their families. These findings highlight negative professional behavior by students who were not exposed to these patients at all and indicate a professional value gap. Students display professional behavior not expected of sophomore nursing students (and in general), and this issue should be examined in depth. Our findings point to the need to expose students to the care of patients in the process of rehabilitation and expand and deepen the knowledge base on this topic. Developing students’ knowledge and providing them with professional opportunities to meet and care for these patients and to encounter the rehabilitation process can promote positive attitudes towards the field, positive feelings and thoughts, and positive professional behavior. These recommendations are enhanced and receive added importance in light of the increase in life expectancy in developed countries [17], the improvement of medical and technological care [18–20], and the increase in patient survival rates after brain events or other events that impair physical, emotional, and daily functioning [17]. The rehabilitation field is becoming increasingly vital and the nurse’s role in rehabilitation is central and essential. Nurses’ central role in the multidisciplinary rehabilitation process has already been documented and found to be associated with the patient’s quality of life and those of the family and caregivers [4, 21, 22]. Therefore, health policymakers and nurse educators should increase and promote the exposure of nursing students to rehabilitation patients and their unique needs through academic courses and the development of clinical skills. Academic training must be made available worldwide, and more scientific studies must be funded to enable evidence-based nursing in rehabilitation [4]. The findings of the current study support this statement.

A significant difference was found in students’ attitudes regarding their perceived capability to care for rehabilitation patients: students who had previous experience with rehabilitation patients in their professional or personal life had a significantly more positive attitude regarding their capability to care for rehabilitation patients than their peers who had no professional or personal previous exposure to rehabilitation, which means that exposure to rehabilitation patients may affect students’ attitudes toward the care of these patients. This finding is congruent with earlier findings among nursing students in other clinical fields, emphasizing the central role of exposure and interaction with complex patients on students’ attitudes. Increasing the quantity and quality of student’s experience can impact their confidence in their knowledge and skills regarding the capability to care for complex patients [12, 23, 24].

With regard to negative professional behavior, only students who had previous exposure to rehabilitation patients in their professional and personal life reported low perceived negative professional behavior, compared to the other two groups. This finding should receive the attention of nurse educators as it points to the need to strengthen and emphasize professional values and professional behavior towards patients. Sümen et al. (2022) found a low positive association between nursing students’ positive professional attitudes and caring behavior [25]. Negative professional behavior toward rehabilitation patients, or any patient, is not the desired professional behavior, and students must be given professional tools to deal with complex rehabilitation patients. Nursing educators must understand the reasons for the negative professional behavior and try to address them. Professional values must be imparted, whereby even in the case of a complex patient [26] with low rehabilitation potential (as in the vignette the students faced in the current study) one must still adhere to high professional values and not neglect the patient’s care. Studies have shown that knowledge and awareness of professional values should be further improved among nursing students worldwide [27, 28]. Acquiring professional values will help nursing students reach professional decisions, including ethical and moral decisions [29]. The current study’s findings reinforce the need to develop higher professional values among sophomore nursing students regarding rehabilitation patients.

Group C participants in the current study, who had been previously exposed to rehabilitation patients and were familiar with rehabilitation patients, were older than the other two groups and expressed lower negative professional behavior toward rehabilitation patients. Similar findings were reported in an integrative literature review, indicating an association between age and the nurse’s professional values. Notably, an increase in age was associated with higher positive professional values. Other factors described in this literature review were the nurse’s level of education and learning activities [30]. In the current study, all students had the same level of education and learning activities. A possible explanation may be that age is essential for displaying lower negative professional behavior toward rehabilitation patients. Nonetheless, previous exposure and engagement in the care of rehabilitation patients on the professional or personal level is another factor not previously explored.

The current study has several limitations related to the research design and tools. It employed a convenience sampling drawn from a single university and was based on self-reports. Although this university has the largest nursing department in Israel, this might limit the generalizability of the findings to the entire population of second-year nursing students. This study could be improved by drawing participants from several universities in the future. The questionnaire included only closed-end questions. Adding more open questions and interviews with students and educators could have provided more information and a deeper understanding of students’ perceptions and comprehension of the subject of rehabilitation.

The sections referring to thoughts and professional behavior considered only positive thoughts and negative professional behavior, as recommended in the original tool. In the future, we recommend adding negative thoughts and positive professional behavior to the tool in order to explore the full range of the phenomena. The vignette used in the current study dealt with one case of nurse-patient-family encounters. Adding more clinical scenarios may uncover different and more detailed findings than found in the current study. In addition, the MAS questionnaire was adapted for rehabilitation patients. Content validation was conducted, and internal consistency was calculated. However, it is recommended to validate the questionnaire in additional statistical ways. Finally, the response rate in our study was less than 50% and there are no details regarding the students who did not respond to the study.

## Conclusions

Students’ exposure to rehabilitation patients and expanding their knowledge base on the subject can promote positive attitudes, feelings, thoughts, and professional behavior toward this field. The research findings highlight the need to expand and deepen students’ knowledge in the field of rehabilitation to promote positive attitudes and strengthen professional values and positive professional behavior. Moreover, nursing professional values and professional behavior toward rehabilitation patients should be refined and strengthened.

### Electronic supplementary material

Below is the link to the electronic supplementary material.

Supplementary Material 1

## Data Availability

The datasets used and/or analyzed during the current study are available from the corresponding author on reasonable request.

## Abbreviations

UHC: Universal health coverage
ICN: International Council of Nurses
ARN: The Association of Rehabilitation Nurses
CBR: Community-based rehabilitation
COM-B: Capability, opportunity, and motivation
