# Supplementary material for: Sophomore nursing students’ perception of their Professional Behavior toward Rehabilitation patients: a cross-sectional study
Source: BMC Nurs. 2023 Nov 23;22:443. doi: 10.1186/s12912-023-01616-y (PMC10666364; doi:10.1186/s12912-023-01616-y)
Supplement: Supplementary file 1 — Supplementary Material 1 [file 12912_2023_1616_MOESM1_ESM.pdf]

Part A

Please complete or circle the appropriate answer:

1. **Study program: 1.** Baccalaureate 2. Premilitary 3. Accelerated for academic
2. **Sex:** 1. Male 2. Female
3. **Age** (in years)
4. **Country of Birth:** a. Israel b. Other
5. **Religion:** 1. Jewish 2. Muslim 3. Christian 4. Druze 5. Other
6. **Religiosity:** 1. Secular 2. Traditional 3. Religious 4. Ultra-Orthodox
7. **Marital status:** 1. In a relationship 2. Not in a relationship at present
8. **Have you ever cared for a rehabilitation patient?** 1. No 2. Yes
9. **Do you have a relative who is in a rehabilitation process?** 1. No 2. Yes

Part B

The following is a list of statements describing nursing practice in rehabilitation. Read each statement and rate your agreement on a scale from 1= don't agree at all to 5=strongly agree

	Don't agree at all	Don't agree	Moderately agree	Agree	Strongly agree
1. I know how to work according to the principles of rehabilitation	1	2	3	4	5
2. I know what it means to include the patient in the rehabilitation process	1	2	3	4	5
3. I know how to motivate the patient to participate in the rehabilitation process	1	2	3	4	5
4. I am able to perform the tasks expected of me as a student in the rehabilitation team	1	2	3	4	5
5. I know what is expected of me as a student in the multi-professional rehabilitation team	1	2	3	4	5
6. I know what the patients' families expect of me	1	2	3	4	5
7. I am able to work with the other members of the multi-professional rehabilitation team	1	2	3	4	5
8. I know the differences between my professional role and the role of the other team members	1	2	3	4	5
9. I am proud of the contribution of nursing to the field of rehabilitation	1	2	3	4	5
10. I think that nursing can have a greater impact in the field of rehabilitation	1	2	3	4	5
11. I need further training/studies on work according to the rehabilitation approach	1	2	3	4	5
12. I need more clinical skills in rehabilitation	1	2	3	4	5
13. I need additional interpersonal and emotional skills in rehabilitation	1	2	3	4	5
14. I need additional skills for work in a multi-professional team	1	2	3	4	5
15. I must learn how to integrate a rehabilitation approach when working in other departments as well	1	2	3	4	5
16. The aim of rehabilitation is to improve and retain optimal functioning	1	2	3	4	5

17. Nursing has a vital role in all phases of rehabilitation in the hospital and in the community	1	2	3	4	5
18. The main principle of rehabilitation is to provide care with the patient and not only for the patient	1	2	3	4	5
19. The rehabilitation process is based on the work of a multi-professional team	1	2	3	4	5
20. The rehabilitation process is directed at helping the patient regain independence	1	2	3	4	5
21. Caring for a patient with a potential for rehabilitation is a professional challenge for me	1	2	3	4	5

Part C- Imagine the following incident:

A post-stroke patient is lying in a hospital rehabilitation department. The patient is paralyzed on the right side of the body, is unable to speak, and cannot move. The patient has two pressure sores on the back and heel. Also, the patient's respiratory condition is unstable and he needs oxygen throughout the day. No progress is evident in the rehabilitation process. His family visits frequently, presenting to the attending nurse and doctor the demand to continue providing rehabilitation treatment. Based on the opinion of a multi-professional team, the nurse in charge of the patient identifies a very low rehabilitation potential. The nurse doesn't know what to do.

People have a variety of emotions when involved in such an incident. The following is a list of possible emotions that may arise before, during, or after this incident. Please indicate in each row how likely it is that this emotion will arise in the nurse, on a scale of 1-5, where 1= "not at all" and 5= "very much".

There are no right or wrong answers in the questionnaire, your feeling is the only decisive factor.

**Nurse's feelings regarding the patient**	**Degree of likelihood**				
	**Not at all**<br>**1**	**2**	**3**	**4**	**Very much**<br>**5**
**1. Rejection**	1	2	3	4	5
**2. Pity**	1	2	3	4	5
**3. Fear**	1	2	3	4	5
**4. Helplessness**	1	2	3	4	5
**5. Anger**	1	2	3	4	5
**6. Guilt**	1	2	3	4	5
**7. Stress**	1	2	3	4	5
**8. Nervousness**	1	2	3	4	5
**9. Concern**	1	2	3	4	5

**10. Despondency/ bad mood**	1	2	3	4	5
**11. Relaxation / Serenity**	1	2	3	4	5
**12. Empathy (for the patient)**	1	2	3	4	5
**13. Empathy (for the family)**	1	2	3	4	5

People have a variety of thoughts when they are involved in such an incident. The following are a list of possible thoughts that could arise before, during or after this incident. Please indicate in each row how likely it is that the thought will occur to the nurse (on a scale of 1-5):

**The nurse's thoughts**	**Degree of likelihood**				
1. **I must take good care of the patient**	**Not at all**<br>**1**	**2**	**3**	**4**	**Very much**<br>**5**
2. **I like to care for people with challenging issues**	1	2	3	4	5
3. **I can explain the patient's true condition to the family**	1	2	3	4	5
4. **If I will be persistent enough, the patient will recover**	1	2	3	4	5
5. **It is important that I talk to the family and explain the patient's condition**	1	2	3	4	5
6. **I will let another nurse take care of the subject**	1	2	3	4	5
7. **It's hard for me to care for such patients**	1	2	3	4	5
8. **I don't have the patience to care for such patients**	1	2	3	4	5
9. **I understand the family's wish**	1	2	3	4	5
10. **There is nothing more I can do with the family**	1	2	3	4	5

People utilize a variety of <u>behaviors</u> when involved in such an incident. The following is a list of possible behaviors that can occur before, during, or after this incident. Please indicate in each row how likely it is that <u>the nurse</u> will behave this way (on a scale of 1-5):

**Behaviors**	**Degree of likelihood**				
	**Not at all**<br>**1**	**2**	**3**	**4**	**Very much**<br>**5**
**1. Try to convince the family to transfer the patient to a regular nursing care department**	1	2	3	4	5
**2. Ignore the family**	1	2	3	4	5
**3. Try to convince the family that the patient cannot continue in rehabilitation**	1	2	3	4	5
**4. Find an excuse to transfer the responsibility to other nurses**	1	2	3	4	5
**5. Find the personal strength to continue caring for the patient**	1	2	3	4	5
**6. Care for the patient unenthusiastically**	1	2	3	4	5
**7. Care for the patient professionally**	1	2	3	4	5
**8. Discuss the issue in a multidisciplinary team**	1	2	3	4	5

Thank you for your cooperation!
